# Minimal Invasive Pancreatoduodenectomy: A Comprehensive Systematic Review and Metanalysis of Randomized Controlled Clinical Trials

**DOI:** 10.1245/s10434-025-16990-x

**Published:** 2025-02-12

**Authors:** Claudio Ricci, Vincenzo D’Ambra, Laura Alberici, Carlo Ingaldi, Margherita Minghetti, Giulia Bonini, Riccardo Casadei

**Affiliations:** 1 Department of Internal Medicine and Surgery (DIMEC), Alma Mater Studiorum, University of Bologna, Bologna, Italy; 2 Division of Pancreatic Surgery, IRCCS Azienda Ospedaliero-Universitaria Di Bologna, Bologna, Italy

**Keywords:** Minimally invasive surgery, Pancreatoduodenectomy, Mortality, Morbidity

## Abstract

**Background:**

The role of a minimally invasive approach (MI) in patients who underwent pancreatoduodenectomy (PD) remained unclear.

**Methods:**

A systematic search of randomized controlled trials was conducted. A random-effects meta-analysis was conducted, reporting risk ratio (RR) or mean difference (MD). The primary endpoints were the morbidity, mortality, and R1 rate. The secondary endpoints were clinically relevant postoperative pancreatic fistula (POPF), postpancreatectomy hemorrhage (PPH), delayed gastric emptying (DGE), biliary fistula, reoperation, length of stay (LOS), time to functional recovery (TFR), and readmission.

**Results:**

The meta-analysis includes seven studies and 1428 patients: 618 (46.5%) in the OPD arm and 711 (53.5%) in minimally invasive pancreaticoduodenectomy (MIPD). The mortality rate was 2.9% for MIPD and 2.6% for OPD (RR 1.11 [range 0.53–2.29]). The major morbidity rate was 29.4% for MIPD and 25.6% for OPD (RR 1.11 [range 0.53–2.29]). The R1 rate was 6.2% for MIPD and 7% for OPD (RR 0.80 [0.54–1.20]). The operative time, comprehensive complication index score, POPF, PPH, DGE, biliary fistula, reoperation, readmission, LOS, TFR, and harvested lymph nodes were similar. Greater than 25% of heterogeneity was observed for major morbidity, operative time, POPF, LOS, TFR, and harvested lymph nodes. No publication bias was registered.

**Conclusions:**

Minimally invasive pancreaticoduodenectomy was not superior to OPD and provided marginal advantages in short-term results. Further efforts should be addressed to clarify the impact of learning curve in MIPD results and the economic sustainability of MIPD, particularly robotic approach.

**Supplementary Information:**

The online version contains supplementary material available at 10.1245/s10434-025-16990-x.

Pancreatoduodenectomy (PD) is the treatment of choice for benign and malignant head pancreatic and periampullary neoplasms.^1^ However, PD remains one of the few surgical procedures for which the minimally invasive approach (MIPD) does not represent the gold standard of care.^2^ Despite the first encouraging data favoring MIPD,^3^ the first randomized controlled trials (RCTs) about the laparoscopic approach (LPD) have not confirmed the advantages versus the open approach (OPD).^4–7^ At least three high-quality studies have suggested that LPD produces marginal advantages compared with OPD, probably irrelevant from the clinical point of view.^8–10^ Another criticism was that LPD implies a considerable operative time and a fatiguing learning curve.^11^ For this reason, the robotic approach (RPD) has been proposed to overcome the limits of LDP.^12,13^ Recently, three new RCTs comparing OPD with RPD have been published, providing robust and direct information about the advantages of the robotic approach.^14–17^ To clarify whether the MIPD approach could be a “game-changer” in pancreatic surgery, an updated meta-analysis was performed to compare MIPD with OPD. The trial sequential analysis (TSA) approach was used to estimate simultaneously the type I and type II errors.^18^

## Materials and Methods

The manuscript was developed following the PRISMA recommendations (Preferred Reporting Items for Systematic Reviews and Meta-Analyses).^19^

### Eligibility Criteria

The eligibility criteria were reported by using the “Population-Intervention-Control-Outcomes-Studies” (PICOS) approach.^20^ The “Population” was patients with head pancreatic neoplasms who received PD. The “Intervention” arms were any MIPD. The “Control” arm was the current standard approach, namely OPD. The studies were included only when reported the morbidity, mortality, and resection rate margin were reported. Only RCTs were included.

### Information Source, Search, Study Selection, and Data Collection Process

The literature review was based on one of the previous meta-analyses, updating them.^7^ The last search was performed on October 26, 2024. The systematic review was conducted on PubMed, Scopus, the ISI-Web of Science, and Cochrane Central Register of Controlled Trials (CENTRAL). The search string was the following: (“robotic surgical procedures”[MeSH Terms] OR “robotic”[Text Word] OR “laparoscopy”[MeSH Terms] OR “laparoscopy”[Text Word]) AND (“pancreaticoduodenectomy”[MeSH Terms] OR “pancreaticoduodenectomy”[Text Word] OR “Whipple”[Text Word] OR “Whipple procedure”[Text Word] OR “pancreatectomy”[Text Word] OR “pancreatic surgery”[Text Word] OR “pancreatic resection”[Text Word]). The research was conducted by translating the string into an appropriate form using the SR accelerator.^21^

### Data Items

The qualitative assessment of the studies was performed by using the revised tool to assess the risk of bias in randomized trials (RoB 2).^22^ Two authors (VD and LA) performed Rob2 evaluation. Any disagreement was solved with a collegial discussion with the first author (CR). Moreover, the following variables were extracted for descriptive purposes: authors, affiliation/country, year of publication, registration number, design, number of surgeons involved, learning curve required, and sample size were described. The primary endpoints were postoperative major morbidity, mortality, and radicality of resection (R1rate). The secondary endpoints were operative time, comprehensive complication index (CCI) clinically relevant postoperative pancreatic fistula (POPF), postpancreatectomy hemorrhage (PPH), delayed gastric emptying (DGE), biliary fistula, reoperation, readmission, length of stay, time to functional recovery (TFR), and harvested lymph nodes.^23–26^ Trial sequential analysis was used to test the false-positive and false-negative results. False-positive results can be excluded when *p* < 0.05; however, (1) the sample size required (RIS) to exclude type I error is reached; (2) when the effect size is too significant (much more than 0.05) to overcome the monitoring boundary. The monitoring boundary represents the *p* value for which the type I error can be excluded even if RIS has not yet been reached. The monitoring boundary values change based on accrued sample size (ASS): the higher the ASS, the lower the value of the monitoring boundary. False-negative results can occur when the RIS to exclude type I is not obtained, but *p* ≥ 0.05.^18^

## Summary Measurements and Methods of the Analysis

The results were described by using risk ratios (RRs) or mean differences (MDs) with 95% confidence intervals (CI). The heterogeneity was measured with the I^2^ and Cochrane Q tests.^27^ Publication/reporting bias was tested by using Egger’s and Begg’s tests.^28^ Metaregression analysis was performed in the presence of nonnegligible heterogeneity (I^2^ > 25% or *p* value of Q test < 0.05) only for critical endpoints.^29^ The analysis was performed by using the netmeta package for R software.

## Results

### Studies Selected

Only three new studies were eligible for the analysis after deduplication and eligibility criteria assessment. Supplementary Fig. 1 shows the PRISMA flowchart.

### Study Characteristics, Network Structures, and Geometries

Table 1 summarizes the characteristics of the seven studies. All studies were registered. Four (57.1%) were conducted in Western countries, and four (57.1%) were multicentric. In four (60%) studies, the intervention arm was LPD, whereas in two, OPD was compared with RPD. In the remaining, both LPD and RPD were considered. Only three studies were patient-blinded. The number of surgeons involved ranged from 1 to 14 per study. A learning curve in both OPD and MIPD procedures was required in all studies, with very different cutoffs. The cumulative conversion rate was 7.7% (range 3.1–23.5%). The sample size was 1428 patients clustered into the three arms: 618 (46.5%) in the OPD arm, and 711 (53.5%) in the MIPD arm.

**Table 1:** Characteristics of the studies included

Study	Affiliation/hospital	Year	Acronyms	Registration	Design	Blinded	Surgeon* (N)	Learning curve (No. MIPD; No. PD )	Conversion rate n/total (%)	Sample size
Palanivelu et al.^3^	Hepatopancreatobiliary Surgery, GEM Hospital and Research Centre, Tamil Nadu India	2016	PLOT	NCT02081131	OPD vs. LPD	No	Yes (2)	Yes (>25; >25)	1/32 (3.1)	64
Poves et al.^4^	Department of Surgery, Hospital del Mar, Barcelona, Spain	2018	PADULAP	ISRCTN93168938	OPD vs. LPD	No	Yes (1)	Yes (-;-)	8/34 (23.5)	61
van Hilst et al.^5^	Netherlands, multicenter	2019	LEOPARD-2	NTR5689	OPD vs. LPD	Patients-blinded	Yes (9)	Yes (>20, >50)	10/50 (20)	99
Wang et al.^6^	China, multicenter	2021	TJDBPS01	NCT03138213	OPD vs. LPD	Patients-blinded	Yes (14)	Yes (104;104)	11/297 (4)	594
Klotz et al.^13^	Department of General, Visceral and Transplantation Surgery, Heidelberg University Hospital, Heidelberg, Germany	2024	EUROPA	DRKS00020407	RPD vs. OPD	No	Yes (2)	Yes (40; 40)	6/26 (23)	62
Liu et al.^14^	China, multicenter	2024	-	ChiCTR2200056809	RPD vs. OPD	No	Yes (3)	Yes (40;60)	3/81 (3.7)	164
De Graaf et al.^15,16^	Europe, multicenter	2024	DIPLOMA-2	ISRCTN27483786	MIPD^§^ vs OPD	Patients-blinded	NA	NA	16/190 (8.4)	288

*N* number, *LPD* laparoscopic pancreaticoduodenectomy, *OPD* open pancreaticoduodenectomy, *RPD* robotic pancreatoduodenectomy, *RoB-2* Revised Cochrane risk-of-bias tool for randomized trials, *NA* not available

^*^Surgeon who performed minimally invasive pancreatoduodenectomy, ^§^90% of procedures were RPD

### Synthesis of Results

#### Primary Endpoints

**Fig. 1:**
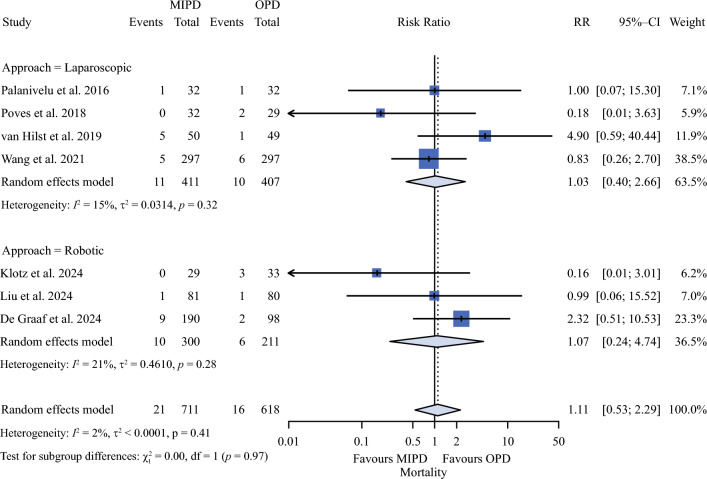
Forest plot for mortality. *OPD* open pancreaticoduodenectomy; *MIPD* minimally invasive pancreaticoduodenectomy; *RR* risk ratio; *CI* confidence interval

**Fig. 2:**
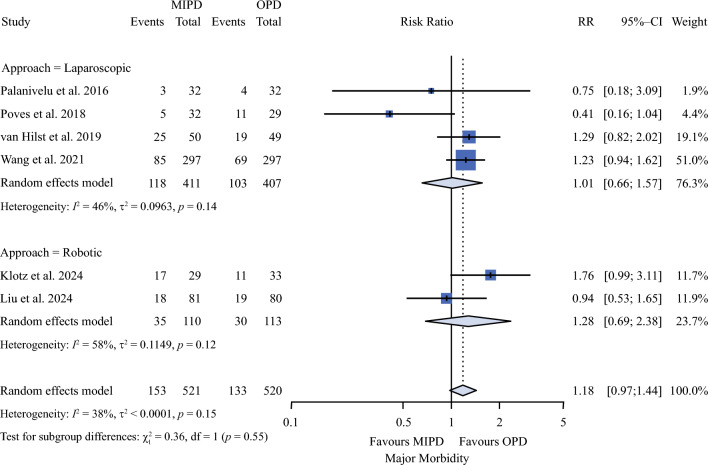
Forest plot for major morbidity. *OPD* open pancreaticoduodenectomy; *MIPD* minimally invasive pancreaticoduodenectomy; *RR* risk ratio; *CI* confidence interval

As reported in Table 2, mortality (Fig. 1) rates were 2.9% and 2.6% in MIPD and OPD groups (RR 1.1; 95% CI 0.53–2.29). Also, major morbidity (Fig. 2) rates were similar between the two groups (29.4% vs. 25.6%; RR 1.18; 95% CI 0.97–1.44). R1 rates were 6.2% and 7% in MIPD and OPD groups (RR 0.80; 95% CI 0.54–1.10).

**Table 2:** Meta-analysis of all outcomes

Outcomes	N (%) or mean (SD)		Effect		Heterogeneity		Publication bias		TSA		
	MIPD	OPD	RR or MD (95% CI)	*p*	I^2^ (%)	*p*-value of Q	Egger^§^	Begg^	ASS	RIS	Error
*Critical*											
Mortality	21/711 (2.9)	16/618 (2.6)	1.11 (0.53 to 2.29)	0.788	2%	0.409	0.482	0.176	1329	163,525	II
Major morbidity	153/521 (29.4)	133/520 (25.6)	1.18 (0.97 to 1.44)	0.092	38%	0.152	0.286	0.573	1041	18,501	II
R1	32/518 (6.2)	36/516 (7)	0.80 (0.54 to 1.20)	0.276	0%	0.723	0.416	0.573	1034	9490	II
*Noncritical*											
Operative time (min)	342 ± 41	308 ± 41	50.91 (-1.47 to103.29)	0.057	98%	<0.001	0.471	0.573	1041	1091	True equivalence
CCI	21 ± 18.5	17.8 ± 20.8	-0.01 (-3.12 to 3.11)	0.997	0%	0.380	0.710	0.601	943	32	True equivalence
CR-POPF	114/711 (16)	109/618 (17.6)	0.87 (0.64 to 1.17)	0.276	29%	0.723	0.656	0.652	1329	24,057	II
PPH (grade B-C)	45/521 (8.6)	50/520 (9.6)	0.87 (0.59 to 1.29)	0.496	0%	0.599	0.759	0.851	1041	6711	II
DGE (grade B-C)	82/521 (15.7)	78/520 (15)	1.05 (0.66 to 1.68)	0.825	54%	0.056	0.777	0.851	1041	2,031,993	II
Biliary fistula	33/521 (6.3)	29/520 (5.6)	1.17 (0.71 to 1.91)	0.535	0%	0.686	0.280	0.189	1041	12,031	II
Reoperation	24/521 (4.6)	28/520 (5.4)	0.90 (0.11 to 3.84)	0.713	0%	0.592	0.490	0.573	1041	12,484	II
Readmission	38/521 (7.3)	33/520 (6.3)	1.13 (0.73 to 1.76)	0.585	0%	0.845	0.961	0.573	1041	10,046	II
LOS (days)	13 ± 4.6	12.4 ± 4.6	-0.50 (-2.44 to 1.43)	0.608	86%	<0.001	0.727	0.652	1329	1825	II
TFR (days)	8.6 ± 4.4	8.9 ± 3.4	0.97 (-1.65 to 3.59)	0.468	94%	<0.001	0.969	0.602	457	1099	II
Harvested lymph-nodes	14 ± 3.0	13.9 ± 2.6	0.73 (-0.37 to 1.83)	0.195	85%	<0.001	0.543	0.851	1041	1151	II

*SD* standard deviation, *MIPD* minimal invasive pancreaticoduodenectomy, *OPD* open pancreaticoduodenectomy, *RR* risk ratio, *MD* mean difference, *CI* confidence interval,* I*^*2*^ heterogeneity, *Q p*-value referred to Q Cochrane, *CCI* comprehensive complication index, *R1* microscopic involvement of resection margin, *CR-POPF* clinically relevant postoperative pancreatic fistula, *PPH* postpancreatectomy hemorrhage, *DGE* delayed gastric emptying, *LOS* length of stay, *TFR* time to functional recovery, *TSA* trial sequential analysis: for dichotomous endpoint the clinical difference was set to relative reduction of 20%; for operative time the minimal clinical difference was set to ±30 min with a SD of ±15 min; for CCI the minimal clinical difference was set to 7 points; for LOS and harvested lymph-nodes the minimal clinical difference was set to 1 day with a SD of 1 day; type II error = false equivalence; type I error = false difference

^§^Egger test for publication bias; ^^^Begg test for publication bias

#### Secondary Endpoint

Operative time seems longer in MIPD than OPD (342 vs. 308 min) with a nonsignificant MD (50.91; 95% CI −1.47 to 103.29). The CCI was similar (21 vs. 17.8) with a MD −0.01 (95% CI −3.12 to 3.11). The POPF, PPH, DGE, and biliary fistula rates were comparable between the two groups with RR of 0.87 (95% CI 0.64–1.17), 0.87 (95% CI 0.59–1.29), and 1.05 (95% CI 0.66–1.68). The reoperation rate was 4.6% versus 5.4% in the MIPD and OPD groups (RR 0.90; 95% CI 0.11–3.84). Readmission rates were 7.3% versus 6.3% in MIPD and OPD (RR 1.13; 95% CI 0.73–1.76). The postoperative stay was similar using LOS (MD −0.50; 95% CI −2.44 to 1.43) and TFR (MD 0.97; 95% CI −1.65 to 3.59). The mean number of harvested lymph nodes was similar (14 vs. 13.9; MD 0.73; 95% CI −0.37 to 1.83).

#### Heterogeneity, Publication Bias, TSA, and Metaregression

Nonnegligible heterogeneity was observed for major morbidity (I^2^ = 38%), operative time (I^2^ = 98%), POPF (I^2^ = 28%), DGE (I^2^ = 54%), LOS (I^2^ = 86%), TFR (I^2^ = 94%), and harvested lymph-nodes (I^2^ = 85%). No publication bias was observed.

**Fig. 3:**
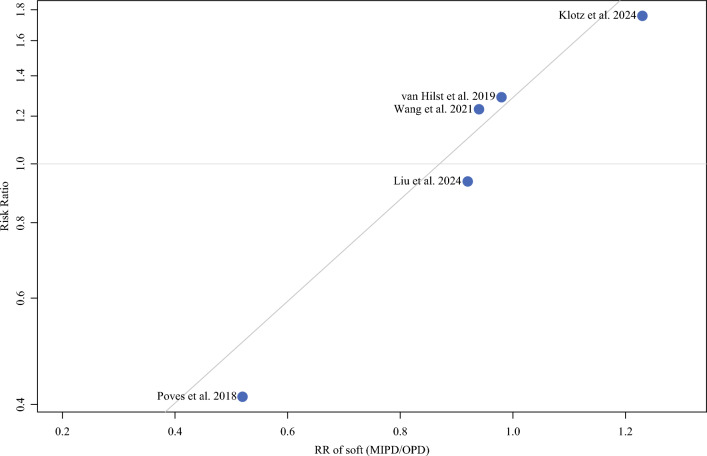
Metaregression for major morbidity and soft pancreas. The x-axis represents the ratio between the proportion of patients with soft pancreas in the MIPD arm and OPD arm; the y-axis was RR for major morbidity

Trial sequential analysis demonstrated that all equivalences were at risk of type II error except for operative time and CCI, which were “true” equivalences. Metaregression showed that a difference in age between the two groups could significantly influence the overall RR for major morbidity (β = 0.42; R^2^ = 100%; *p* = 0.001). Adjusted RR for major morbidity was 1.1 (range 0.90–1.10). Also, the distribution of soft pancreas (Fig. 3) influenced the RR for major morbidity rate (β = 1.94; R^2^ = 100%; *p* = 0.001) and adjusted RR was 1.28 (95% CI 1.04–1.58 CI), suggesting an increased risk for MIPD. The rate of PDAC between the two arms significantly influenced the morbidity rate (β = 1.28; R^2^ = 100%; *p* = 0.021), and the adjusted RR was 1.18 (95% CI 0.97–1.45).

## Discussion

This study demonstrates that routinary use of the minimally invasive approach for PD remains did not provide any advantages. Randomized controlled trials did not confirm the hypothetical advantages of robotic and laparoscopic PD.^30^

Moreover, the TSA approach demonstrated that new RCTs could be useless, at least for critical endpoints, and any attempt to demonstrate the superiority of MIPD is time-consuming and doomed to fail. Indeed, several patients should be randomized to demonstrate that MIPD is as safe as OPD, because the differences between the approaches were so slight that the number of patients required to find some differences or demonstrate the equivalence was unrealistic. For this reason, it is reasonable to affirm that the controlled data disown the promising role of MIPD as a game changer. Moreover, even if MIPD safety is not entirely and definitively demonstrated, it seems unrealistic that the minimally invasive approach can reduce the significant morbidity and mortality rate in PD. Thus, adopting MIPD in clinical practice should pass through the demonstration of other clear advantages. However, data available from this meta-analysis did not demonstrate any sure short-term advantages. One of the main arguments for promoting minimally invasive surgery was the reduction of postoperative stay. However, in pancreatic surgery, hospital stay was mainly related to the occurrence of major morbidity rate, which, despite the minimally invasive approach, remained high.^31^ Moreover, length of stay was not the main goal of pancreatic surgery, in which safety and long-term results are the parameters to monitor the quality of care.

Minimally invasive pancreaticoduodenectomy, particularly RPD, implies another new problem in pancreatic surgery. Data obtained from the paper of Klotz et al.^14^ suggested that adopting a robotic platform cost 12,073 euros more for patients. Gaber et al.^32^ indicated increased costs also using LPD. When a new procedure is introduced, when the advantages are not clear, and when the cost increase is inevitable, the sustainability of the procedure should be studied.^33^ However, it is easy to imagine the results of a cost-effectiveness study: if the increasing cost is sure, a gain in survival seems too optimistic. The survival and quality of life of patients who underwent PD mainly depended on their oncological status.^33^ There are currently no reasons to believe that MIPD could impact the disease-free or overall survival of PDAC.

Finally, conceding that an increase in survival was possible using a different surgical approach, even if it seems too optimistic, the question of a fatiguing learning curve remains an open problem.^34^ The learning curve of LPD seems unrealistic, as well as for several high-volume surgical centers, requiring nearly 100 procedures for surgeons.^11^ The learning curve of RPD seems to be shorter even if proficiency is realized in 250 procedures.^35^ In other words, in minimally invasive pancreatic surgery, the risk is that several patients could receive a suboptimal treatment before obtaining some marginal advantages for others without significantly impacting life expectancy and with significant costs for the healthcare system. Moreover, different strategies seem promising to reduce the problems related to the learning curve, such as the opportunity to learn directly from a procto or nationwide training program for the second generation of robotic surgeons.^36,37^

The present study has some limitations. The included studies covered a long period during which changes occurred. Another limitation was the lack of a standardized definition of some relevant outcomes. Moreover, despite the randomized design, some data collection has a specific quote of subjective measurement that is not corrigible with within-study bias evaluation. These biases can be contained by using metaregression, which permits the interpretation of heterogeneity but does not eliminate it. Also, the wide conversion rate range represents a limit for this study. Even if all authors declared a completed learning curve, at least three studies presented a conversion rate greater than 20%, reducing the externalization of the results.^4,5,13^ We tried to mitigate this bias by using metaregression analysis, which did not demonstrate a significant impact on the results. Finally, there needs to be a more direct comparison between LPD and RPD, even if subanalysis and metaregression did not suggest an advantage of the robotic approach.

## Conclusions

The present study suggests that both MIPD have marginal advantages compared with OPD. Despite the optimistic conclusion of several retrospective comparative studies, neither LPD nor RPD have the role of the game changer in performing PD. Indeed, despite the randomized design, neither data quality nor an apparent beneficial effect supported the routinary use of a minimally invasive approach for PD. Moreover, the cost, particularly for RPD, is very high for the quality of life gained by the patients and for the resources of the healthcare system. The further randomization of patients in MIPD arms, if aimed at demonstrating superiority for the safety of short-term efficacy, could be useless because it seems indemonstrable.

## Supplementary Information

Below is the link to the electronic supplementary material.

Supplementary file1 (DOCX 53 KB)
