# Supplementary material for: Minimal Invasive Pancreatoduodenectomy: A Comprehensive Systematic Review and Metanalysis of Randomized Controlled Clinical Trials
Source: Ann Surg Oncol. 2025 Feb 12;32(5):3614–22. doi: 10.1245/s10434-025-16990-x (PMC11976793; doi:10.1245/s10434-025-16990-x)
Supplement: Supplementary file 1 — Supplementary file1 (DOCX 53 KB) [file 10434_2025_16990_MOESM1_ESM.docx]

**Previous studies**

**Identification of new studies via databases and registers**

Studies included in previous version of review (n =4)

Records identified from*:

Medline/Pubmed (n=671)

Web of Science (n=400)

Cochrane Central Register of Controlled Trials (CENTRAL)

(n=0)

Scopus (n=889)

Total (n=1,960)

Records removed *before screening*:

Duplicate records removed (n =919)

**Identification**

Total studies included in review

(n = 6)

Reports assessed for eligibility

(n = 97)

Records screened

(n =1,041)

Records excluded**

(n =917)

**Screening**

Reports excluded:

Meta-analysis (n = 39)

Other topics or non-randomized design (n = 56)

New studies included in review

(n =2)

**Included**

*Consider, if feasible to do so, reporting the number of records identified from each database or register searched (rather than the total number across all databases/registers).

**If automation tools were used, indicate how many records were excluded by a human and how many were excluded by automation tools.

*From:*  Page MJ, McKenzie JE, Bossuyt PM, Boutron I, Hoffmann TC, Mulrow CD, et al. The PRISMA 2020 statement: an updated guideline for reporting systematic reviews. BMJ 2021;372:n71. doi: 10.1136/bmj.n71

For more information, visit: <http://www.prisma-statement.org/>
